# Self-Assembled Nanoscaled Metalloporphyrin for Optical Detection of Dimethylmethylphosphonate

**DOI:** 10.1155/2019/7689183

**Published:** 2019-03-18

**Authors:** Mingbo Wu, Hongsheng Yang, He Wei, Xueli Hu, Bo Qu, Mei Chen

**Affiliations:** ^1^Department of Biomedical Sciences, Chengdu Medical College, Chengdu, Sichuan 610500, China; ^2^The First Affiliated Hospital of Chengdu Medical College, Chengdu, Sichuan 610500, China

## Abstract

The self-assembly approach has been widely adopted in the effort to design and prepare functional materials. Herein, we report the synthesis and optical properties of metalloporphyrin nanoparticles. Nanoscaled particles of 5,10,15,20-tetraphenylporphyrin manganese (MnTPP) and 5,10,15,20-tetraphenylporphyrin indium (InTPP) were produced in the water/dimethylsulfoxide (DMSO) mixed solution by self-assembly approach. The absorbance intensity at the characteristic peak of the monomeric and nanoscaled metalloporphyrins decreased when they interact with dimethylmethylphosphonate (DMMP). Detection limits of MnTPP and InTPP nanoparticles to DMMP were 10^−9^ and 10^−10^ L/L, respectively, and detection limits of monomeric MnTPP and InTPP to DMMP were 10^−6^ and 10^−7^ L/L, respectively. Density functional theory (DFT) calculations on MnTPP and InTPP with DMMP as axial ligands had been performed in the B3LYP/6-31g (d) approximation. Their optimized geometries and binding energies were found to depend very strongly on the central metal ion, and InTPP was more sensitive for DMMP detection in contract to MnTPP. All the experimental and theoretical results demonstrated that nanoscaled metalloporphyrin have potential prospects in determination for public safety.

## 1. Introduction

Nanoscaled materials have many unique properties, such as surface effects, small size effects, and macroscopic quantum tunneling effects, which are important features in many technological applications [1–3]. Porphyrins are particularly attractive building blocks for nanoscaled materials because of its intimate packing of aromatic macrocycles. In particularly, metalloporphyrins have stronger axial coordination effect than nonmetallic porphyrins due to the addition of metal ions [4]. Thus, it ensures metalloporphyrins have richer photophysical and photochemical properties than nonmetallic porphyrins [5, 6]. In previous works, it had demonstrated that porphyrin nanoparticles were more efficient catalysts than porphyrin monomer [7]. The unique properties of nanoparticles containing the free base or closed-shell porphyrins can be exploited for sensors and displays [8, 9].

Sarin is a deadly toxic chemical warfare agent (CWA). It acts on nervous system by inhibiting the activity of cholinesterase, causing the accumulation of acetylcholine [10]. Even a low level sarin may lead to headache, rapid twitching of voluntary muscles, abdominal cramps, and death [11, 12]. The rapid and sensitive determination of sarin in trace level has been a hot area of research for several years [13]. Researches on its detection are always processed by using compounds with similar chemical structure, i.e., DMMP [14, 15]. Traditional methods for the detection of DMMP, including chromatography and spectroscopic analysis, are sensitive and reliable, but also are tedious and time-consuming [16–18]. Accordingly, to realize rapid warning and field deployment, sensors for facilitating the task of on-site monitoring of nerve agents are high desirable [19]. Currently, the enzyme inhibition sensors can be used for DMMP monitoring [20–22]. But, due to the extreme condition (optimum temperature and pH value) requirements for enzymes, the enzyme inhibition sensors still have some limitations in practical utilization. Thus, a novel sensitive material is required for DMMP monitoring.

**Figure 1:**
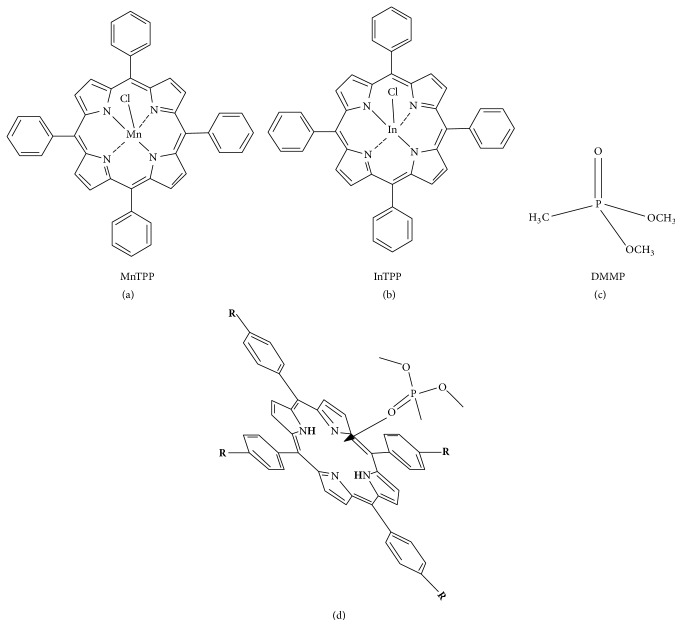
Molecular structure of MnTPP (a), InTPP (b), and DMMP (c) and schematic diagram of porphyrin combined with DMMP (d).

Since the rich coordination chemistry can be used in chemical-sensor applications by detecting changes induced in their physicochemical properties by adding axial ligands, porphyrins have been wildly used for the detection of hazardous, toxic gases [23, 24], environment pollutant nitroaromatic compounds [25], insecticides, and pesticides [26]. It has demonstrated that the presence of individual organic molecules strongly affects the spectrophotometric characteristics of porphyrins [27]. Our previous works indicated that both monosulfonate tetraphenylporphyrin (TPPS_1_) and meso-tetra(4-sulfonatophenyl) porphyrin (TPPS) can be used to detect DMMP due to the spectral changes associated with the interactions between porphyrins and DMMP. Accordingly, since the excellent performance of nanoscaled materials, herein, we firstly prepare the functional self-assembled nanoscaled MnTPP and InTPP and present the results of spectroscopic study for DMMP detection (Figure 1). And we try to elucidate the electronic structures of MnTPP and InTPP with DMMP as axial ligands for understanding their mechanism of interactions, with the help of the DFT in the B3LYP/6-31g (d) approximation.

## 2. Experimental 

### 2.1. Apparatus and Reagents

The finer details about surface structure of nanoparticles were observed by using Nova 400 nano-FE-SEM. Optical spectra of nanoparticles are characterized by UNICO UV 2100 spectrophotometer using a quartz cell. Ultrapure water for aggregation of MnTPP and InTPP was purified by a water purification system (Millipore, made in France) to the resistivity of ~18.2 MΩ cm. IKA RH digital KT/C magnetic stirrer was used in the preparation process.

MnTPP and InTPP were obtained from Frontier Scientific (Logan, UT, USA) and were stored refrigerated in the dark for use. DMMP was purchased from Sigma (St. Louis, MO, USA). Dimethyl sulfoxide (DMSO) was purchased from Kelong Chemical Agent Factory (Chengdu, China). Ultrapure water was generated by a Millipore Direct-Q Water system (Molsheim, France). All chemicals are analytical grade and were used without further purification.

### 2.2. Preparation of Nanoscaled MnTPP and InTPP

100 uL 0.125g/L stored solution of MnTPP and InTPP was injected in 5 mL ultrapure water at optimum temperature 50°C, respectively. We had already investigated under stirring with rotating speed 1200 rpm 10 minutes. Finally, a transparent colloid disperse solution was obtained.

### 2.3. FE-SEM Characterization of Nanoscaled MnTPP and InTPP

Fresh solution of MnTPP and InTPP nanoparticles was spin coated on conductive glass which was immersed in DMSO dilute solution for 3 days and then dried 5 days in dark at room temperature until liquid disappears. Finally, a porphyrin film was obtained and we characterized it by using FE-SEM.

### 2.4. Determination to Trace Amounts of DMMP

1mL transparent colloid was extracted into a test tube, and then 1 mL DMMP solution with different volume concentration was injected, respectively. The volume concentration of DMMP solution was 1.0 × 10^−5^ L/L, 1.0 × 10^−6^ L/L, 1.0 × 10^−7^ L/L, 1.0 × 10^−8^ L/L, 1.0 × 10^−9^ L/L, and 1.0 × 10^−10^ L/L, respectively. After fully reaction a new solution was obtained, we characterized it by using UV-Vis spectrophotometer. Certainly, we also compared solution reacted from DMMP and MnTPP and InTPP monomer solution under the same reaction conditions.

### 2.5. DFT Calculations

All calculations were performed using Gaussian 03 software [28]. By using density functional theory and B3LYP functional with the basis set 6-31G(d) for C, H, O, and N atoms, 6-311G(d, p) basis sets for P and Cl atoms and the pseudopotential Lanl2dz for Mn and In. Stationary points were confirmed to be minima or transition states by calculating the normal vibrations within the harmonic approximation. All DFT-computed relative energies are corrected for zero-point vibrational energies (ZPE).

## 3. Result and Discussion

### 3.1. FE-SEM Analysis of Nanoscaled MnTPP

**Figure 2:**
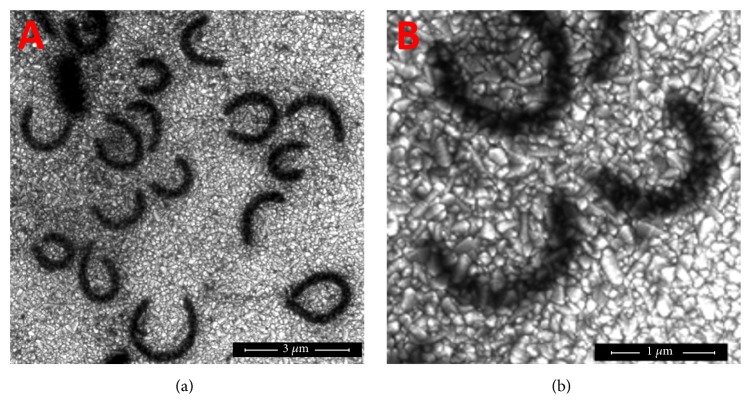
FE-SEM images with two magnifications of MnTPP nanoparticles.

The self-assembly approach has been widely adopted in the effort to design and prepare functional materials. The FE-SEM images of nanoscaled MnTPP are shown in Figure 2. The black rings are nano-MnTPP and small bright white particles are conductive glass substrate. It suggests that the morphology of well-distributed nano-MnTPP is cricoid or half-cricoid and the average width of the rings is approximately 200 nm. To our knowledge, the arrangement of macrocycles in aggregates generally fall in into two types, “J” (edge-to-edge) and “H” (face-to-face) interactions. Herein, we proposed that nano-MnTPP were made up of J-type aggregates, which were mainly powered by the noncovalent interactions^.^

**Figure 3:**
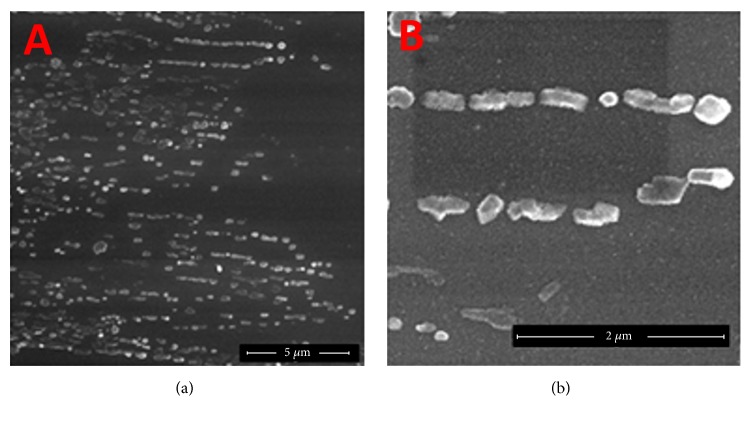
FE-SEM images with two magnifications of InTPP nanoparticles.

As shown in Figure 3, the FE-SEM images of nano-InTPP suggested that the morphology of well-distributed nano-InTPP is rodlike and the average width of the particles is approximately 150 nm. These phenomena indicate that InTPP nanoparticles were made up of J-type aggregates.

### 3.2. Optical Properties of Two Porphyrin Monomers and Their Nanoparticles

**Figure 4:**
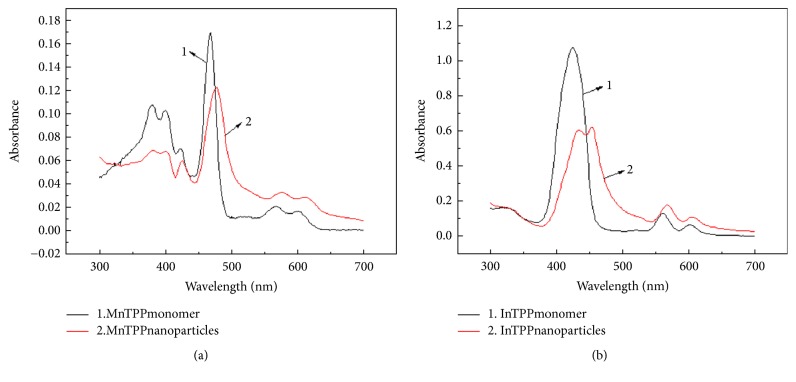
UV-Vis spectra of MnTPP monomer and MnTPP nanoparticles (a); InTPP monomer and InTPP nanoparticles (b).

MnTPP monomer had a strong absorption peak at 468 nm, known as Soret absorption band, also called B band (Figure 4(a)). This band belongs to the second electron excited state of *π* → *π*^*∗*^ transition and it is generated by transition of porphyrin a1*μ*(NHOMO, *π*)→eg (LUMO, *π*^*∗*^). There were two weak absorption peaks at 568 and 602 nm respectively called Q band, which belongs to the first electron excited state of *π* → *π*^*∗*^ transition and it is generated by transition of porphyrin a 2*μ* (HOMO, *π*) →eg (LUMO, *π*^*∗*^). The shape and location of the Soret band shift obviously larger than Q band, and therefore we discussed Soret band and its nearby areas emphatically in this paper. The UV-Vis spectra of porphyrin nanoparticles are significantly different compared to the spectra of the corresponding porphyrin solutions. Comparing with the MnTPP monomer, the absorption spectra of nano-MnTPP are found to be broadened at 477 nm, and the Soret band of the MnTPP nanoparticles has an obvious red shift. The absorption value of the nano-MnTPP is much smaller too.  Figure 4(b) also indicates the B band of InTPP monomer has a strong absorption peak at 424 nm. However, the absorption spectra of nano-InTPP are found to be broadened and it is split into two reduced and red shifted bands at 433 nm and 453 nm, respectively.

In general, the arrangement of macrocycles in aggregates generally falls into two types: “J” (edge-to-edge) interactions are characterized by red shifts and “H” (face-to-face) interactions are characterized by blue shifts. Thus, the optical spectra suggest the main drive mechanism of the two nanoparticles is “J” interactions. Currently, both types of interactions in the porphyrin nanoparticles are well understood to be indicative of electronic coupling of the chromophores [29]. The red shift of absorption spectra may be explained by frontier molecular orbital theory [30]. The HOMO-LUMO energy gap trends to increase as the particle diameter decrease. Accordingly, the internal stress of nanomaterials increases with the decrease of particle diameter (p=2*ϒ*/r, p represents internal stress, *ϒ* represents surface tension, and r represents particle radius). This causes the band structure changing, the overlap of electronic wave function increasing, the structure space between energy levels decreasing, HOMO-LUMO energy gap decreasing, and thus a red shift occurs. This UV-Vis spectrum of the nanoscaled porphyrin suggests that the red shifted bands are indicative of electronic coupling of the J-type aggregate structure [31].

### 3.3. UV-Vis Spectra Analysis of MnTPP and InTPP Monomer Reacts with DMMP

**Figure 5:**
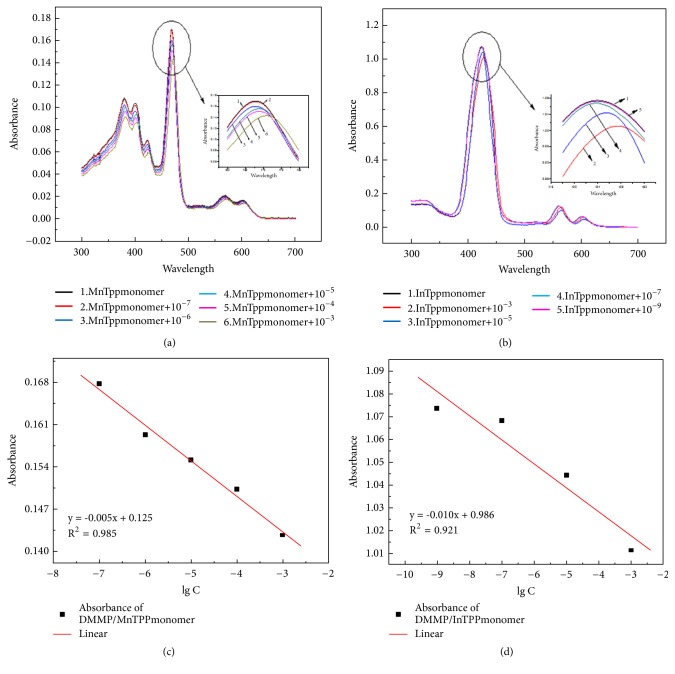
UV-Vis spectra of MnTPP monomer (a) and InTPP monomer (b) react with different concentration DMMP; logarithmic curve of max-absorbance values of MnTPP monomer (c) and InTPP monomer (d) reacts with DMMP.

In this study, before the effects of the self-assembled nanoscaled porphyrin were established, the spectral changes associated with the interactions between porphyrins and DMMP should be ensured firstly. It has demonstrated that DMMP can combine with porphyrin molecule through interactions of hydrogen bond, Van der Waals force, etc. [32, 33]. These interactions produce the conformational changes of porphyrin molecule and further contribute to an obvious decrease of the UV-visible absorption spectrum. As shown in Figure 5(a), with the concentration of DMMP increasing, the peaks of Soret band are characterized by red shift and its absorbance value decreases gradually. In particularly, the spectrophotometric characteristics indicate that detection limit of MnTPP monomer for DMMP is approximately 1.0 × 10^−6^ L/L. There are several electron donor groups in DMMP molecule, e.g., methoxy groups (Figures 1(c) and 1(d)). Therefore, the DMMP molecules are combined with MnTPP, causing the HOMO-LUMO energy gap of porphyrin macrocycle reducing, and characterized as the red shift of absorption peaks.

Similar to that of MnTPP, the characteristic peaks of Soret band of InTPP were red shifted and the absorbance value decreases gradually with the concentration of DMMP increasing (Figure 5(b)). When the concentration of DMMP is 1.0 × 10^−7^ L/L, the absorption spectrum of solution after reaction is almost the same as blank sample of MnTPP monomer. It indicates that detection limit of InTPP monomer for DMMP is approximately 1.0 × 10^−7^ L/L.

### 3.4. UV-Vis Spectra Analysis of MnTPP and InTPP Nanoparticles Reacts with DMMP

**Figure 6:**
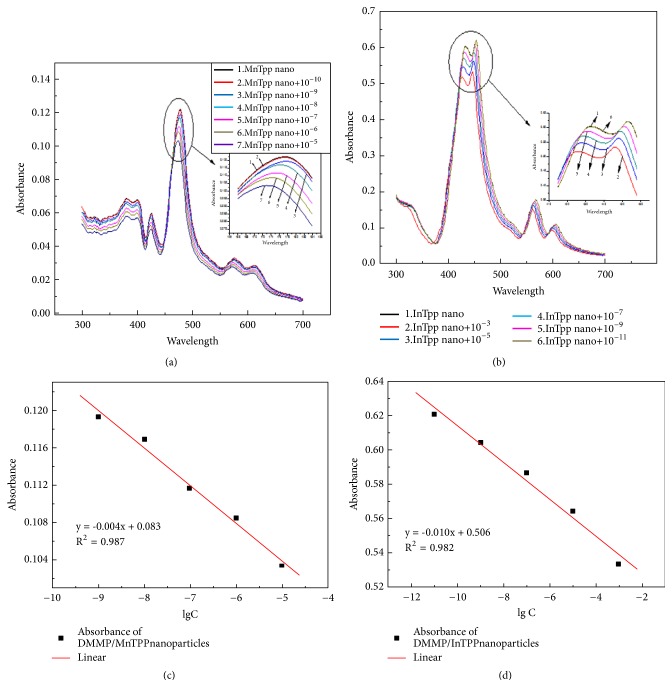
UV-Vis spectra of MnTPP-nano (a) and InTPP-nano (b) react with different concentration DMMP; logarithmic curve of max-absorbance values of MnTPP-nano (c) and InTPP-nano (d) reacts with DMMP.

Porphyrin nanoparticles were more efficient catalysts than porphyrin monomer; thus they can be exploited for sensors and displays. As shown in Figure 6(a), after MnTPP nanoparticles reacting with DMMP, the characteristic peak of Soret band shows a blue shift comparing with that of MnTPP monomer. Moreover, the extent of blue shift increases over the concentration of DMMP rising. The blue shift of absorption peaks suggests the increase of space between structure energy levels and HOMO-LUMO energy gap. Thus, it decreases the overlap of electronic wave function and reduces the surface tension and internal stress of nanomaterials. Considering the enhancement of conjugation on porphyrin macrocycle, we propose that DMMP may play a role of connector among nanoparticles in these events. With concentration of DMMP increasing, the absorbance value of characteristic peaks of Soret band decreases gradually, which corresponds with that of monomer. In particularly, the detection limit of nano-MnTPP for DMMP is approximately 1.0 × 10^−9^ L/L. Comparing with the detection limit of MnTPP monomer for DMMP, it is improved by three orders of magnitude.

Similar to the UV-Vis spectra of nanoscaled MnTPP, Figure 6(b) shows that the characteristic peaks of Soret band of InTPP nanoparticles were blue shifted after reacting with DMMP. Moreover, the extent of blue shift increases over the concentration of DMMP rising. Thus, we proposed that DMMP may play a role of connector among nanoparticles. Meanwhile, consistent with the monomer, the absorbance value of characteristic peaks of Soret band decreases gradually with concentration of DMMP increasing. And the detection limit of InTPP nanoparticles for DMMP is approximately 1.0 × 10^−10^ L/L. Comparing with the detection limit of InTPP monomer for DMMP, it is improved by three orders of magnitude.

### 3.5. DFT Calculations

**Figure 7:**
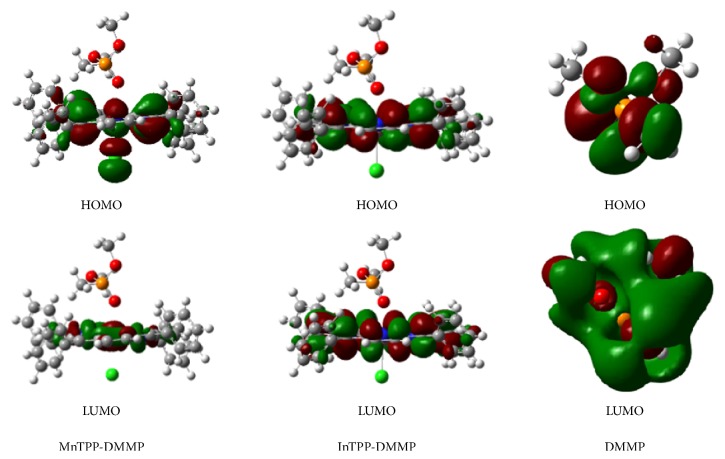
HOMO photos and LUMO photos of MnTPP-DMMP, InTPP-DMMP, and DMMP.

It is known that axial ligation strongly affects the redox and photovoltaic properties of metal porphyrins [34, 35]; e.g., iron porphyrins with coordinating axial ligands are diamagnetic (S=0) compared with four-coordinate species (S=1) [36, 37]. Thus, elucidating the electronic structure of metal porphyrins with axial ligands is very important for understanding their biochemical functions. Using the method of the density functional theory in the B3LYP/6-31g (d) approximation, the geometry of MnTPP and InTPP with DMMP as axial ligands was fully optimized as shown in Figure 7. The optimized geometries of MnTPP and InTPP are found to depend strongly on the central metal ion. Meanwhile, according to the hybridized electron orbit of porphyrin-DMMP, DMMP donates its electrons to the porphyrin macrocycle because of conjugated effect. Thus, the interaction can be explained by the combined action of *σ*-donation and *π*-back-donation mechanism; DMMP donates electrons to form *π* electron conjugated system, causing the HOMO-LUMO energy gap of porphyrin macrocycle reducing.

Computational investigations have been wildly used in investigating molecular electrical transport properties of regular porphyrins and several derivatives, including the calculations of energy gaps of HOMO-LUMO, dipole moment, and the binding energy [38]. As shown in Table 1, the frontier orbital gap of MnTPP is obviously larger than that of InTPP, which suggests InTPP has a much greater affinity for DMMP. And the binding energy represents the stability of complex. Since the binding energy of MnTPP-DMMP is smaller than that of InTPP-DMMP, it shows that MnTPP binds weakly to DMMP in contrast to InTPP. Thus, the calculated results suggest InTPP is more sensitive for DMMP detection, which have excellent correlation between theoretically and experimentally derived UV-visible spectra. It also proved that B3LYP/6-31G (d) computational method was well suited to metalloporphyrin molecules.

## 4. Conclusions

The results presented here provide a simple alternative method for the detection of CWA simulant DMMP by monitoring the absorbance spectral changes of the nanoscaled metalloporphyrin. The morphology of well-distributed nano-MnTPP was cricoid or half-cricoid and the average width of the rings is approximately 200 nm, and the InTPP nanoparticles were rodlike and the average width of the rings is approximately 150 nm. Although the absorption spectra of the monomeric and nanoscaled metalloporphyrins exhibited a significant shift to DMMP, the detection limits of MnTPP and InTPP nanoparticles to DMMP were 10^−9^ and 10^−10^ L/L, respectively, and detection limits of MnTPP and InTPP nanoparticles to DMMP were 10^−6^ and 10^−7^ L/L, respectively.

Meanwhile, the DMMP complex with MnTPP and InTPP had been investigated theoretically with DFT functional (B3LYP). The optimized geometries suggested DMMP combined with porphyrin as axial ligands, and the central metal ion strongly affected their hybridized electron orbit. The calculated energy gaps of MnTPP and InTPP and binding energy to DMMP suggested InTPP was more sensitive for DMMP detection. These results had excellent correlation between theoretically and experimentally derived UV-visible spectra, which proved that B3LYP/6-31G(d) functional was well suited to metalloporphyrin molecules. As a result, nanoscaled metalloporphyrin has potential application for the detection of trace amounts DMMP, as well as optical sensors for CWAs.

## Figures and Tables

**Table 1:** Calculated energy gaps of MnTPP and InTPP (Δ*E*_HOMO-LUMO gap_) and binding energy (*E*_bind_) to DMMP.

	Δ*E*_HOMO-LUMO gap_ (kJ mol^−1^)	*E*_bind_ (kJ mol^−1^)
MnTPP	224.086	19.563
InTPP	199.341	26.862

## Data Availability

The data used to support the findings of this study are available from the corresponding author upon request.

## References

[1] Chen M., Hou C., Huo D., Bao J., Fa H., Shen C. (2016). An electrochemical DNA biosensor based on nitrogen-doped graphene/Au nanoparticles for human multidrug resistance gene detection. *Biosensors and Bioelectronics*.

[2] Kluková L., Bertok T., Kasák P., Tkac J. (2014). Nanoscale-controlled architecture for the development of ultrasensitive lectin biosensors applicable in glycomics. *Analytical Methods*.

[3] Komori K., Terse-Thakoor T., Mulchandani A. (2015). Bioelectrochemistry of heme peptide at seamless three-dimensional carbon nanotubes/graphene hybrid films for highly sensitive electrochemical biosensing. *ACS Applied Materials & Interfaces*.

[4] Nakagaki S., Mantovani K. M., Machado G. S., De Freitas Castro K. A. D., Wypych F. (2016). Recent advances in solid catalysts obtained by metalloporphyrins immobilization on layered anionic exchangers: A short review and some new catalytic results. *Molecules*.

[5] Hitzenberger J. F., Dammann C., Lang N. (2016). Making the invisible visible: Improved electrospray ion formation of metalloporphyrins/-phthalocyanines by attachment of the formate anion (HCOO(-)). *Analyst*.

[6] Beletskaya I., Tyurin V. S., Tsivadze A. Y., Guilard R., Stern C. (2009). Supramolecular chemistry of metalloporphyrins. *Chemical Reviews*.

[7] Zhang L., Peng D., Liang R.-P., Qiu J.-D. (2015). Graphene quantum dots assembled with metalloporphyrins for "turn on" sensing of hydrogen peroxide and glucose. *Chemistry - A European Journal*.

[8] Penza M., Alvisi M., Rossi R. (2011). Carbon nanotube films as a platform to transduce molecular recognition events in metalloporphyrins. *Nanotechnology*.

[9] Ryabova V., Schulte A., Erichsen T., Schuhmann W. (2005). Robotic sequential analysis of a library of metalloporphyrins as electrocatalysts for voltammetric nitric oxide sensors. *Analyst*.

[10] de Lima W. E. A., Francisco A., da Cunha E. F. F. (2017). Mechanistic studies of new oximes reactivators of human butyryl cholinesterase inhibited by cyclosarin and sarin. *Journal of Biomolecular Structure and Dynamics*.

[11] Herkert N. M., Lallement G., Clarençon D., Thiermann H., Worek F. (2009). Comparison of the oxime-induced reactivation of rhesus monkey, swine and guinea pig erythrocyte acetylcholinesterase following inhibition by sarin or paraoxon, using a perfusion model for the real-time determination of membrane-bound acetylcholinesterase activity. *Toxicology*.

[12] Pohanka M., Karasova J. Z., Kuca K. (2010). Colorimetric dipstick for assay of organophosphate pesticides and nerve agents represented by paraoxon, sarin and VX. *Talanta*.

[13] Climent E., Biyikal M., Gawlitza K. (2016). A rapid and sensitive strip-based quick test for nerve agents tabun, sarin, and soman using BODIPY-modified silica materials. *Chemistry - A European Journal*.

[14] Zhao Y., He J., Yang M. (2009). Single crystal WO(3) nanoflakes as quartz crystal microbalance sensing layer for ultrafast detection of trace sarin simulant. *Analytica Chimica Acta*.

[15] Trusso Sfrazzetto G., Millesi S., Pappalardo A. (2017). Nerve Gas Simulant Sensing by a Uranyl–Salen Monolayer Covalently Anchored on Quartz Substrates. *Chemistry - A European Journal*.

[16] Bonnot K., Cuesta-Soto F., Rodrigo M. (2014). Biophotonic ring resonator for ultrasensitive detection of DMMP as a simulant for organophosphorus agents. *Analytical Chemistry*.

[17] Wild A., Winter A., Hager M. D., Schubert U. S. (2012). Fluorometric, water-based sensors for the detection of nerve gas G mimics DMMP, DCP and DCNP. *Chemical Communications*.

[18] Wang Y., Zhou Z., Yang Z., Chen X., Xu D., Zhang Y. (2009). Gas sensors based on deposited single-walled carbon nanotube networks for DMMP detection. *Nanotechnology*.

[19] Jang S., Kim J., Koh Y., Ko Y. C., Woo H.-G., Sohn H. (2007). Multi-encoded rugate porous silicon as nerve agents sensors. *Journal of Nanoscience and Nanotechnology*.

[20] Mills C. E., Obermeyer A., Dong X., Walker J., Olsen B. D. (2016). Complex Coacervate Core Micelles for the Dispersion and Stabilization of Organophosphate Hydrolase in Organic Solvents. *Langmuir*.

[21] Chen C., Hu S.-Y., Luo D.-Q., Zhu S.-Y., Zhou C.-Q. (2013). Potential antitumor agent from the endophytic fungus Pestalotiopsis photiniae induces apoptosis via the mitochondrial pathway in HeLa cells. *Oncology Reports*.

[22] Gurton K. P., Felton M., Dahmani R., Ligon D. (2007). In situ infrared aerosol spectroscopy for a variety of nerve agent simulants using flow-through photoacoustics. *Applied Optics*.

[23] Ossato A., Vigolo A., Trapella C. (2015). JWH-018 impairs sensorimotor functions in mice. *Neuroscience*.

[24] Li X., Liang J., Hou Z., Zhu Y., Wang Y., Qian Y. (2014). Coordination complex pyrolyzation for the synthesis of nanostructured GeO(2) with high lithium storage properties. *Chemical Communications*.

[25] Wiesner S., Wagner A., Hübner O., Kaifer E., Himmel H.-J. (2015). Thermochromism of Cu(I) tetrakisguanidine complexes: reversible activation of metal-to-ligand charge-transfer bands. *Chemistry - A European Journal*.

[26] D'Auria I., Lamberti M., Mazzeo M., Milione S., Roviello G., Pellecchia C. (2012). Coordination chemistry and reactivity of zinc complexes supported by a phosphido pincer ligand. *Chemistry - A European Journal*.

[27] Johnson B. J., Melde B. J., Thomas C. (2010). Fluorescent silicate materials for the detection of paraoxon. *Sensors*.

[28] Azarias C., Pawelek M., Jacquemin D. (2017). Structural and optical properties of subporphyrinoids: a TD-DFT study. *The Journal of Physical Chemistry A*.

[29] Wang Z., Medforth C. J., Shelnutt J. A. (2004). Self-metallization of photocatalytic porphyrin nanotubes. *Journal of the American Chemical Society*.

[30] Gong X., Milic T., Xu C., Batteas J. D., Drain C. M. (2002). Preparation and characterization of porphyrin nanoparticles. *Journal of the American Chemical Society*.

[31] Huang C., Li Y., Yang J., Cheng N., Liu H., Li Y. (2010). Construction of multidimensional nanostructures by self-assembly of a porphyrin analogue. *Chemical Communications*.

[32] Wang Y., Jia W., Strout T., Ding Y., Lei Y. (2009). Preparation, characterization and sensitive gas sensing of conductive core-sheath TiO(2)-PEDOT nanocables. *Sensors*.

[33] Schwalb N. K., Temps F. (2009). A modified four-state model for the "dual fluorescence" of N 6,N6-Dimethyladenine derived from femtosecond fluorescence spectroscopy. *The Journal of Physical Chemistry A*.

[34] Ohta T., Liu J.-G., Nagaraju P., Ogura T., Naruta Y. (2015). A cryo-generated ferrous-superoxo porphyrin: EPR, resonance Raman and DFT studies. *Chemical Communications*.

[35] Hong Z., Wert J., Asher S. A. (2013). UV resonance raman and DFT studies of arginine side chains in peptides: insights into arginine hydration. *The Journal of Physical Chemistry B*.

[36] Pandith A. H., Islam N. (2014). Electron transport and nonlinear optical properties of substituted aryldimesityl boranes: a DFT study. *PLoS ONE*.

[37] Krawczyk P. (2010). DFT study of linear and nonlinear optical properties of donor-acceptor substituted stilbenes, azobenzenes and benzilideneanilines. *Journal of Molecular Modeling*.

[38] Cárdenas-Jirón G. I., Barboza C. A., López R., Menéndez M. I. (2011). Theoretical study on the electronic excitations of a porphyrin-polypyridyl ruthenium(II) photosensitizer. *The Journal of Physical Chemistry A*.

